# Recent Updates on Outbreaks of Shiga Toxin-Producing *Escherichia coli* and Its Potential Reservoirs

**DOI:** 10.3389/fcimb.2020.00273

**Published:** 2020-06-04

**Authors:** Jun-Seob Kim, Moo-Seung Lee, Ji Hyung Kim

**Affiliations:** ^1^Infectious Disease Research Center, Korea Research Institute of Bioscience and Biotechnology, Daejeon, South Korea; ^2^Department of Biomolecular Science, KRIBB School of Bioscience, Korea University of Science and Technology (UST), Daejeon, South Korea; ^3^Environmental Diseases Research Center, Korea Research Institute of Bioscience and Biotechnology, Daejeon, South Korea

**Keywords:** Shiga toxin-producing *Escherichia coli*, Shiga toxin, STEC reservoir, HUS, environmental transmission

## Abstract

Following infection with certain strains of Shiga toxin-producing *Escherichia coli* (STEC), particularly enterohemorrhagic ones, patients are at elevated risk for developing life-threatening extraintestinal complications, such as acute renal failure. Hence, these bacteria represent a public health concern in both developed and developing countries. Shiga toxins (Stxs) expressed by STEC are highly cytotoxic class II ribosome-inactivating proteins and primary virulence factors responsible for major clinical signs of Stx-mediated pathogenesis, including bloody diarrhea, hemolytic uremic syndrome (HUS), and neurological complications. Ruminant animals are thought to serve as critical environmental reservoirs of Stx-producing *Escherichia coli* (STEC), but other emerging or arising reservoirs of the toxin-producing bacteria have been overlooked. In particular, a number of new animal species from wildlife and aquaculture industries have recently been identified as unexpected reservoir or spillover hosts of STEC. Here, we summarize recent findings about reservoirs of STEC and review outbreaks of these bacteria both within and outside the United States. A better understanding of environmental transmission to humans will facilitate the development of novel strategies for preventing zoonotic STEC infection.

## Introduction

*Escherichia coli* is a component of the normal flora in the human gut, but some strains are pathogenic. Based on its pathotypes, intestinal pathogenic *E. coli* can be classified into six groups: Shiga toxin (Stx)-producing [STEC, also referred to as verocytotoxin-producing (VTEC) or enterohemorrhagic (EHEC)], enterotoxigenic (ETEC), enteropathogenic (EPEC), enteroaggregative (EAEC), enteroinvasive (EIEC), and diffusely adherent (DAEC) (Kaper et al., 2004). Among those, STEC tends to be a clonal group characterized by somatic (O) antigen, and more than 200 serotypes of *E. coli* have been known to produce Stxs based on their molecular and genetic features. In addition, a new classification scheme of five seropathotypes (A–E) based on virulence, serological and genetic features has been suggested due to the various symptoms and severity of clinical STEC infections (Frankel et al., 1998; Nataro and Kaper, 1998; Boerlin et al., 1999; Karmali et al., 2003). However, a recent massive outbreak in Germany raised questions about the efficacy of this categorization because the strain involved was not classified as type A or B based on its genetics (specifically, it was negative for the LEE Island). Hence, in this review, we summarize outbreaks and STEC isolates by serotype, not seropathotype, based on surveillance reports.

Stxs are a family of bacterial exotoxins expressed by *Shigella dysenteriae* serotype 1 and STEC (Fraser et al., 1994; Sandvig, 2001). These toxins are primary virulence factors responsible for bloody diarrheal disease that can progress to life-threatening systemic sequelae, such as an acute renal failure syndrome (also known as hemolytic uremic syndrome, HUS), as well as central nervous system (CNS) abnormalities (Tarr et al., 2005; Lee et al., 2016; Lee and Tesh, 2019). The toxins produced by STEC are classified as type 1 (Stx1) and type 2 (Stx2), and several Stx1/Stx2 subtypes and variants have been reported based on the receptor preference and toxin potency (Scheutz et al., 2012; Melton-Celsa, 2014). And among those, Stx2, which is more potent than Stx1, causes clinically severe weight loss and renal injury (Lentz et al., 2011; Pradhan et al., 2016).

Multiple studies have focused on revealing the source and transmission route of STEC infections in humans and the food chain (Erickson and Doyle, 2007; Kintz et al., 2017). Animals are undoubtedly the most important carriers of STEC, as these strains have been isolated from a wide variety of domestic and human-associated animal species (Persad and LeJeune, 2014; Espinosa et al., 2018). Several lines of evidence have confirmed zoonotic human infections caused by contact with companion and domestic animals (Chalmers et al., 1997; Luna et al., 2018). In addition, work in recent decades has emphasized the importance of wildlife surveillance, as a large proportion of emerging zoonotic pathogens are of wildlife origin (Jones et al., 2013), and increasing numbers of wild animals have been shown to be potential STEC reservoirs (Espinosa et al., 2018). Although the need for the One Health approach has been continuously emphasized in STEC research, surveillance studies have generally been limited to domestic animals (Garcia et al., 2010). However, a recent STEC surveillance study revealed that more distantly related fields, such as aquaculture, should be included as important areas of interest and monitored accordingly. In this review, we update the list of animal species recently reported as STEC reservoirs. In so doing, our goal is to emphasize the importance of applying the interdisciplinary One Health approach in surveillance systems by strengthening multi-sectorial collaboration between agriculture, aquaculture, and wildlife science, as well as to provide a broad perspective on industrial fields relevant to food production.

## STEC Global Outbreaks and Clinical Isolates

**Figure 1:**
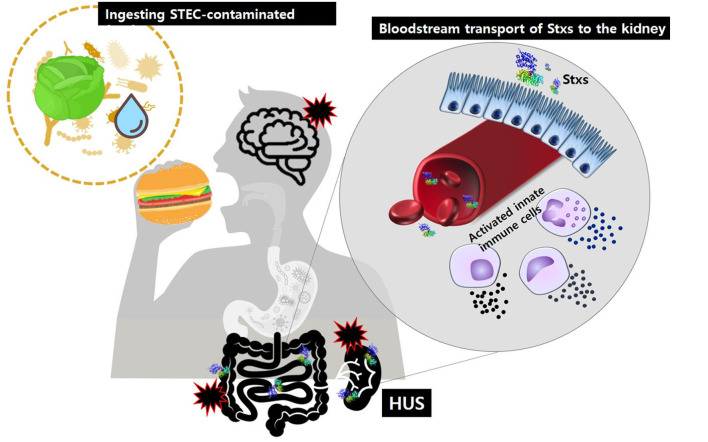
After ingestion of food or water contaminated with pathogenic STEC, Stxs may cross the intestinal epithelial barrier via M-cell uptake and transcytosis or paracellular transport. Once in the submucosa, the toxins activate innate immune cells, such as neutrophils or monocytes that act as “carrier” cells to deliver Stxs in the bloodstream and may also further exacerbate tissue injury via localized production of proinflammatory cytokines. Ultimately, the toxins are transferred to glomerular endothelial cells and tubular epithelial cells, which are rich in the toxin receptor Gb3. Damage to the kidney, the primary target organ, leads to D + HUS (diarrhea-associated hemolytic uremic syndrome).

Historically, Stxs and verotoxin were studied separately. Stxs was discovered by Kiyoshi Shiga in 1898 as a factor involved in bacterial dysentery caused by *S. dysenteriae* serotype I (Kaper and O'Brien, 2014). Independently, in 1977, verotoxin was discovered by Konowalchuck in diarrheagenic *E. coli* strains (Konowalchuk et al., 1977). In 1983, Johnson et al. confirmed that two toxins belonged to the same family (Johnson et al., 1983), and they began to be considered together in studies of the first STEC outbreak strains from 1982. Shiga toxin-producing bacteria, including STEC and *S. dysenteriae* serotype 1, are agents of hemorrhagic colitis, which can progress to potentially lethal complications, such as bloody diarrhea-associated HUS (D + HUS) with acute renal dysfunction (Figure 1) and CNS disorders, such as seizure or paralysis. Investigations of major outbreaks have focused on STEC, rather than on *S. dysenteriae* serotype 1 because STEC infections are more common in the broader community than *Shigella* infections.

### In the United States

In 1982, two severe outbreaks that caused HUS occurred in Oregon and Michigan. *E. coli* O157:H7 was isolated from the stool specimens of patients and determined to be the cause of disease (Centers for Disease, 1982). After a year, production of Stxs was confirmed by comparing toxins purified from *S. dysenteriae* and three *E. coli* isolates from the outbreaks (O'Brien et al., 1983). Since then, STEC O157 has rapidly emerged as a major problem in the food industry and clinics. In the 30 years since the first report, a total of 740 outbreaks caused by STEC O157:H7 and O157:NM were reported in the United States. A total of 13,526 cases resulted in 2,765 hospitalizations (20%), 653 HUS (4.8%), and 73 deaths (0.5%) (Rangel et al., 2005; Heiman et al., 2015). In all years since 1994 except for 1997, the annual outbreak size rose above 30 cases a year.

Food is the best-known transmission route of STEC O157. The frequency of foodborne outbreaks has increased dramatically over the past three decades: 183 out of a total of 350 outbreaks (52%) in the first 20 years (1982–2002) vs. 255 out of a total of 390 outbreaks (65%) in the last 10 years (2003–2012). Over the same period, the incidence of outbreaks via other routes has decreased: person-to-person (14–10%), water (9–4%), and other or unknown reasons (21–11%). Interestingly, STEC outbreaks due to animal contact have also become more common, from 11 (3%) in the first 20 years to 39 (10%) in the last 10 years, indicating that animal resources represent important STEC reservoirs (Rangel et al., 2005; Heiman et al., 2015) (see Environmental Transmission section).

Although STEC O157 was the first *E. coli* strain involved in Stx–related disease and remains the most important strain in this regard, non-O157 STEC strains also represent a major public health concern. The Centers for Disease Control and Prevention estimates that 265,000 STEC infections occur each year in the United States, of which STEC O157 causes 36%; thus 64% of STEC infections are non-O157 (Scallan et al., 2011). More than 50 non-O157 STEC serogroups are involved in human illness. The first US outbreak of non-O157 STEC, caused by STEC O111, was reported in 1990; over the next 20 years (1990–2010), 46 outbreaks caused 1,727 illnesses, 144 hospitalizations, and one death. As with O157, food (*n* = 20, 43%) is a major transmission route in non-O157 outbreaks (Luna-Gierke et al., 2014).

Since the first outbreak in 1990, 11 serotypes and one undetermined type have been observed in non-O157 outbreaks. The most commonly isolated serotype is O111, followed by O26; together, O111 and O26 account for more than 60% of outbreaks (Brooks et al., 2005; Luna-Gierke et al., 2014). O103, O121, O45, O145, O104, O165, O69, O84, and O141 are also frequently isolated from outbreak patients. Interestingly, although non-O157 infection is almost twice as common as O157 infection, non-O157 strains cause fewer outbreaks than O157 (Scallan et al., 2011). This might be due to the greater severity of O157 (more hospitalization) or issues with subtyping techniques (e.g., it is difficult to subtype non-O157 strains) (Gould et al., 2013).

### Outside the United States

The World Health Organization (WHO) estimated that STEC infection caused more than 1 million illnesses and 100 deaths in 2010 (Havelaar et al., 2015). Between 1998 and 2016, the European region (EUR) and Western Pacific region (WPR) reported 211 STEC outbreaks (EUR: 176, WPR: 35), far fewer than the number of outbreaks in the Americas (708) (FAO/WHO, 2018).

The largest O157 STEC outbreak ever recorded occurred in the WPR (Japan, 1996) (Fukushima et al., 1999). Of 12,680 symptomatic patients, 121 (0.95%) developed HUS, and three died. After that massive outbreak, the frequency of STEC cases increased dramatically: from 1999 to 2012, more than 3,000 cases were reported in Japan, whereas during the previous 5 years (1991–1995) the annual average was only 105 cases. Following O157, the most frequent serotype, other common serogroups of STEC are O26, O111, O103, O121, and O145 (Terajima et al., 2014).

The most severe outbreak of non-O157 STEC (O104) occurred in EUR (Germany, 2011): over a 3 months period, 3,816 cases were reported. Despite the smaller number of cases relative to the Sakai outbreak, the rates of HUS (*n* = 845, 22.4%) and death (*n* = 54) made the German outbreak historic (Frank et al., 2011). According to surveillance reports from Food- and Waterborne Diseases and Zoonoses and the European Centre for Disease Prevention and Control, the total number of confirmed STEC infections was 3,573 (doi: 10.2903/j.efsa.2011.2090) in 2009, increasing dramatically to 6,073 cases in 2017 (https://doi.org/10.2903/j.efsa.2018.5500). As in other regions, the most commonly reported serogroup from 2009 to 2017 was O157, followed by O26, O103, O91, O145, and O146. However, the proportion of O157 dropped from 51.7 to 31.9%, whereas the proportion of non-O157 infections increased accordingly. Among the 31 countries in Europe, Germany and the United Kingdom had the highest human STEC infection rates.

## Environmental Transmission of STEC

Over the past decade, interest in zoonotic pathogens of wildlife origin has increased because those pathogens were shown to constitute the primary source (>60%) of emerging infectious diseases (Jones et al., 2008). Moreover, adaptation of certain urban exploiter animal species has increased contact between wild animals and humans, potentiating the transmission of zoonotic pathogens by fecal contamination of agri-food, the environment, or the water chain (Rothenburger et al., 2017). Although most *E. coli* are commensal organisms of both humans and animals, the emergence of STEC has been reported in almost all parts of the world and from a wide variety of animal species, including mammals, birds, amphibians, fish, and invertebrates (Persad and LeJeune, 2014; Espinosa et al., 2018). We have updated the list of animal species reported to be reservoir or spillover hosts for, or to be contaminated by, STEC strains (Table 1).

**Table 1:** Animal species recently identified as potential STEC reservoirs.

**Common name**	**Scientific name**	**References**
**MAMMALS**		
**RUMINANTS**		
Cattle	*Bos taurus*	Gyles, 2007
Goats	*Capra aegagrus hircus*	Beutin et al., 1993
Sheep	*Ovis aries*	Gyles, 2007
Water buffalo	*Bubalus bubalis*	Galiero et al., 2005
White-tailed deer	*Odocoileus virginianus*	Sargeant et al., 1999
Red deer	*Cervus elaphus*	Bardiau et al., 2010
Fallow deer	*Dama dama*	Bardiau et al., 2010
Roe deer	*Capreolus capreolus*	Bardiau et al., 2010
American bison	*Bison bison*	Reinstein et al., 2007
Elk	*Cervus canadensis*	Franklin et al., 2013
Llamas	*Lama glama*	Mohammed Hamzah et al., 2013
Alpaca	*Lama pacos*	Leotta et al., 2006
Yak	*Bos grunniens*	Leotta et al., 2006
Eland	*Taurotragus oryx*	Leotta et al., 2006
Antelope	*Antilope cervicapra*	Leotta et al., 2006
Mountain goat	*Oreamnos americanus*	Chandran and Mazumder, 2013
Guanaco	*Lama guanicoe*	Mercado et al., 2004
Moose	*Alces alces*	Nyholm et al., 2015
Chamois	*Rupicapra rupicapra*	Hofer et al., 2012
Ibex	*Capra ibex*	Hofer et al., 2012
**MONOGASTRICS**		
Domestic swine	*Sus domesticus*	Gyles, 2007
Feral swine (or wild boar)	*Sus scrofa*	Wacheck et al., 2010
Horses	*Equus ferus caballus*	Hancock et al., 1998
Donkey	*Equus africanus asinus*	Chandran and Mazumder, 2013
Dogs	*Canis lupus familiaris*	Beutin et al., 1993
Cats	*Felis catus*	Beutin, 1999
Coyote	*Canis latrans*	Chandran and Mazumder, 2013
Fox	*Vulpes vulpes*	Chandran and Mazumder, 2013
Rabbit	*Oryctolagus cuniculus*	Pritchard et al., 2001
Hares	*Lepus timidus*	Espinosa et al., 2018
Pika	*Ochotona daurica*	Espinosa et al., 2018
Raccoon	*Procyon lotor*	Shere et al., 1998
Rats	*Rattus* spp.	Nielsen et al., 2004
Norway rats	*Rattus novegicus*	Cizek et al., 2000
Ground hog	*Marmota monax*	Chandran and Mazumder, 2013
Patagonian cavy	*Dolichotis patagonus*	Leotta et al., 2006
Agouti	*Dasyprocta* spp.	Espinosa et al., 2018
Lowland paca	*Cuniculus paca*	Espinosa et al., 2018
Bear	Unknown	Vasan et al., 2013
Opossum	Unknown	Espinosa et al., 2018
Armadillo	Unknown	Espinosa et al., 2018
Cougar	*Puma concolor*	Espinosa et al., 2018
Macaques	*Macaca* spp.	Espinosa et al., 2018
Peccary	Unknown	Espinosa et al., 2018
Ferrets	*Mustela putorius furo*	Woods et al., 2002
Mice	*Mus* spp.	Wadolkowski et al., 1990
**BIRDS**		
Chicken	*Gallus gallus domesticus*	Ferens and Hovde, 2011
Domestic duck	*Anas platyrhynchos domesticus*	Koochakzadeh et al., 2015
Turkeys	*Meleagris gallopavo*	Ferens and Hovde, 2011
Pigeon	*Columba livia*	Foster et al., 2006
Starling	*Sturnus vulgaris*	Kobayashi et al., 2009
Geese	*Branta canadensis*	Kullas et al., 2002
Turtle dove	*Streptopelia turtur*	Kobayashi et al., 2009
Barn swallow	*Hirundo rustica*	Kobayashi et al., 2009
Cockatiels	*Nymphicus hollandicus*	Gioia-Di Chiacchio et al., 2018
Budgerigars	*Melopsittacus undulatus*	Gioia-Di Chiacchio et al., 2018
Red-legged seriema	*Cariama cristata*	Borges et al., 2017
Roadside hawk	*Rupornis magnirostris*	Borges et al., 2017
Cattle egrets	*Bubulcus ibis*	Fadel et al., 2017
House crows	*Corvus splendens*	Fadel et al., 2017
Moorhens	*Gallinula chloropus*	Fadel et al., 2017
House teals	*Anas crecca*	Fadel et al., 2017
Great egrets	*Ardea alba*	De Oliveira et al., 2018
Lesser Kestrel	*Falco naumanni*	Koochakzadeh et al., 2015
Indian peafowl	*Pavo cristatus*	Milton et al., 2019
Sarus crane	*Antigone antigone*	Milton et al., 2019
Barn swallow	*Hirundo rustica*	Kobayashi et al., 2009
Seagulls	Unknown	Makino et al., 2000
**FISH**		
Nile tilapia	*Oreochromis niloticus*	Cardozo et al., 2018
African sharptooth catfish	*Clarias lazera*	Hussein et al., 2019
Flathead gray mullet	*Mugil cephalus*	Hussein et al., 2019
Atlantic lizardfish	*Synodus saurus*	Hussein et al., 2019
Red porgy	*Pagrus pagrus*	Hussein et al., 2019
Catla	*Labeo catla*	Sekhar et al., 2017
Grass carp	*Ctenopharyngodon idella*	Siddhnath et al., 2018
Mrigal	*Cirrhinus mrigala*	Siddhnath et al., 2018
Common carp	*Cyprinus carpio*	Siddhnath et al., 2018
**AMPHIBIANS**		
Red-eyed tree frog	*Agalychnis callidryas*	Dipineto et al., 2010
Oriental fire-bellied toad	*Bombina orientalis*	Dipineto et al., 2010
**INVERTEBRATES**		
Blue/Mediterranean mussel	*Mytilus edulis/galloprovincialis*	Gourmelon et al., 2006
Pacific oyster	*Crassostrea gigas*	Gourmelon et al., 2006
Common cockle	*Cerastoderma edule*	Gourmelon et al., 2006
Indian white shrimp	*Fenneropenaeus indicus*	Surendraraj et al., 2010
European flat oyster	*Ostrea edulis*	Martin et al., 2019
House fly	*Musca domestica*	Alam and Zurek, 2004
Dung beetle	*Catharsius molossus*	Xu et al., 2003
Black dump fly	*Hydrotaea aenescens*	Szalanski et al., 2004

### Domestic Animals Are Indisputable Reservoirs of STEC

Ruminants are recognized as principal reservoirs of STEC, especially O157 (Gyles, 2007; La Ragione et al., 2009). As with humans, ruminants are exposed to STEC through contaminated feed and drinking water, or by exposure to the feces of other animals that are shedding the bacteria (LeJeune et al., 2001; Persad and LeJeune, 2014). Among ruminants, cattle (especially ruminating post-weaning calves and heifers) are considered to be the most important STEC reservoirs without symptomatic colonization (Caprioli et al., 2005; Gyles, 2007; Ferens and Hovde, 2011). The natural absence of vascular receptors (globotriaosylceramide) in the intestinal vasculature of the cattle inhibits endocytosis and transportation of Stxs to other organs that might be sensitive to the toxins, resulting in asymptomatic colonization in the large intestine (Pruimboom-Brees et al., 2000; Naylor et al., 2003; Nguyen and Sperandio, 2012). Like cattle, smaller ruminants, such as sheep and goats are also recognized as significant carriers due to their ability to harbor STEC O157 and other serotypes; these animals are important asymptomatic shedders in the epidemiology of bacterial infections in the United States, Australia, and Europe (Beutin et al., 1993; Cortes et al., 2005; Gyles, 2007; La Ragione et al., 2009; Brandal et al., 2012). Also as in cattle, the asymptomatic nature of STEC colonization in smaller ruminants might be due to their lack of vascular receptors for Stx (Persad and LeJeune, 2014). In addition, STEC O157 and non-O157 strains have been reported in other domestic or captive ruminant species, such as alpacas, antelopes, American bison, various deer species, elk, llamas, moose, water buffalo, and yaks (Galiero et al., 2005; French et al., 2010; Chandran and Mazumder, 2013; Mohammed Hamzah et al., 2013; Nyholm et al., 2015).

Several recent surveillance studies have provided strong evidence that monogastric farm animals should now be considered as important reservoir or spillover hosts of STEC. Although the prevalence of STEC O157 and other serotypes varies in swine (Fairbrother and Nadeau, 2006; Ferens and Hovde, 2011), pigs have been shown to harbor and shed STEC for up to 2 months post-infection (Booher et al., 2002). Moreover, because pigs possess Stx-sensitive vascular receptors (globotetraosylceramide) in their intestines, they are susceptible to STEC strains possessing Stx2e, which cause edema with apparent clinical signs and mortality (Waddell et al., 1998; Pruimboom-Brees et al., 2000; Fratamico et al., 2004; Steil et al., 2016). Moreover, although horses are not considered reservoirs for STEC due to its low prevalence in that species (Hancock et al., 1998; Pritchard et al., 2009; Lengacher et al., 2010), some cases of clinical infection from equine contact have been reported (Chalmers et al., 1997; Luna et al., 2018); therefore, horses should be considered as a potential source of infection. Domestic poultry, such as chicken, duck, and turkeys have also been reported to carry STEC (Doane et al., 2007; Ferens and Hovde, 2011; Koochakzadeh et al., 2015). In particular, chickens which were experimentally inoculated with STEC O157 can harbor and shed the bacteria in their feces for almost a year (Schoeni and Doyle, 1994).

The importance of companion animals (pets) in the epidemiology of STEC infection should not be underestimated. Via their feces, pets, such as dogs and cats can serve as asymptomatic shedders in the epidemiology of a wide range of STEC serotypes (Beutin, 1999; Roopnarine et al., 2007; Hogg et al., 2009; Rumi et al., 2012). Accordingly, several clinical infections due to canine and feline exposure have been reported (Busch et al., 2007; Persad and LeJeune, 2014; McFarland et al., 2017). STEC has also been found from the feces of wild canids but not felids (Mora et al., 2012; Persad and LeJeune, 2014).

### Wild Animals Are Important Reservoir or Spillover Hosts of STEC

The number of STEC outbreaks associated with the consumption of fruits and vegetables contaminated with wild animal feces is increasing (World Health Organization, 2016). Hence, from a global public health standpoint, it is important to investigate the prevalence of STEC in urban exploiter and wild animals that can transmit the bacteria to human by direct and/or indirect contact. Therefore, several studies have investigated the prevalence of STEC among urban exploiter species, such as rats (Himsworth et al., 2015), pigeons (Gargiulo et al., 2014; Murakami et al., 2014), and flies (Kobayashi et al., 1999; Alam and Zurek, 2004; Keen et al., 2006). In fact, rodents are capable of harboring and shedding STEC, and various serogroups have been recovered from animals living in urban areas and farms (Blanco Crivelli et al., 2012; Kilonzo et al., 2013). Moreover, many wild bird species found in close proximity to livestock operations, waste disposal landfill sites, and human habitation areas have also been identified as potential sources of STEC infection (Cizek et al., 2000; Pedersen and Clark, 2007). In addition, houseflies can harbor and transmit STEC O157 to other animals, demonstrating that insects can be important vectors in the dissemination of STEC within the environment (Kobayashi et al., 1999; Alam and Zurek, 2004; Keen et al., 2006). Because domestic animal feed represents an easy food source for rodents, birds, and insects, these animals are attracted to farms and may transmit STEC between livestock and humans or vice versa.

Likewise, wild animals residing in close proximity to livestock facilities can be contaminated (or harbored) with STEC (Espinosa et al., 2018). Several recent studies emphasized the urgent need to investigate the prevalence of STEC in wild animals, as some large STEC outbreaks were closely related to or originated from contamination from wild animal feces (Laidler et al., 2013; Crook and Senior, 2017; Soderqvist et al., 2019). Although wild animals were identified as a source of STEC in the 1990s, more than 70% of relevant studies were published since the turn of the century, and an increasing number of wild animal species have been identified as reservoir or spillover hosts for STEC (Espinosa et al., 2018). Nevertheless, very little published research has addressed the role of wild animals in the transmission of STEC to humans, domestic animals, and within the food chain. Animals, such as wild boars, deer, birds, and rodents might be involved in direct interspecies contact between humans, domestic, and wild animals, thereby creating a circle of transmission that increases the prevalence of STEC. These species should be thoroughly monitored, as they could potentially cause a spillover or spillback to humans and other animals (Daszak et al., 2000).

### Emerging Reservoirs of STEC and Needs for the One Health Approach

Numerous studies have reported both O157 and non-O157 STEC in fresh fish and shellfish, and their ready-to-eat products in retail markets (Thampuran et al., 2005; Surendraraj et al., 2010; Prakasan et al., 2018), suggesting that human activities, such as handling, processing, and ingestion of the products might be a major source of STEC contamination. Interestingly, fish and shellfish residing in coastal areas, some cultured fish, and those in close proximity to or downstream of animal livestock facilities have been found to be contaminated with STEC (Gourmelon et al., 2006; Sekhar et al., 2017; Cardozo et al., 2018; Siddhnath et al., 2018; Hussein et al., 2019). These results strongly indicate that fish and shellfish are a potential reservoir or spillover hosts of STEC, and that effluent water from STEC-contaminated culture ponds might also be an additional potential source of transmission, emphasizing the need for further investigations of the aquaculture industry.

Based on the findings of recent surveillance approaches, a wide range of domestic, captive, and wild animals, including aquatic animals, can transmit STEC to humans directly by ingestion or contact at farms and petting zoos, or indirectly through fecal contaminations in water sources, vegetable fields, or meats and milks. Moreover, STEC is closely associated with human activities; therefore, the broad expansion of human activities due to technological advances will expand contaminations to an increasingly wider variety of wild organisms and foodstuffs in the future. Therefore, a detailed identification of the prevalence of STEC in various animal species will be essential for epidemiological investigations and the development of proper risk mitigation strategies (Persad and LeJeune, 2014). The integration of human and animal health was appreciated in ancient times, but this idea was comprehensively revisited through the One Health perspective, which proposes a unification of human and veterinary medicine to protect against zoonotic pathogens (King et al., 2008; Zinsstag et al., 2011). Investigations of STEC outbreaks in humans also clearly demonstrate the relevance of the One Health concept (Jay et al., 2007; Laidler et al., 2013; McFarland et al., 2017). Moreover, the importance of a One Health approach for control or prevention of STEC infection has already been emphasized in practical cases (Garcia et al., 2010). A number of new animal species, including those of aquatic origin, have been identified as unexpected reservoir or spillover hosts of STEC. Therefore, we propose an alternative One Health approach in which coordinated multidisciplinary efforts integrate terrestrial and aquatic animal medicine within future STEC surveillance. These efforts should facilitate the development of novel strategies to prevent, control, and treat zoonotic STEC infections.

## Conclusion

Since the advent of systematic and efficient diagnostic techniques, reports of national STEC outbreaks have increased dramatically. The current world-wide surveillance system reveals the impact of STEC infection, the diversity of STEC, and sources of contamination. Although contaminated food is the most prominent source of STEC outbreaks, infections caused by contact with animals has increased over the past 10 years. Hence, understanding of animals as potential STEC reservoir and their transmission is essential for preventing the occurrence of STEC infections and outbreaks. Multiple complex studies aimed at discovering numerous STEC in the various animals have revealed a wide range of strains capable of producing Stxs, however, it remains to be determined to what extent these newly identified reservoirs are involved in the pathogenesis and transmission of the bacteria. In particular, several animals in more distantly related fields, such as fish produced by the aquaculture industry and a wide range of underestimated wild animal species have been reported as potential STEC reservoirs. Therefore, we propose an alternative One Health approach in which coordinated multidisciplinary efforts integrate terrestrial and aquatic animal medicine in the context of future STEC surveillance efforts.

## Author Contributions

Written and revised by J-SK, M-SL, and JK.

## Conflict of Interest

The authors declare that the research was conducted in the absence of any commercial or financial relationships that could be construed as a potential conflict of interest.
